# The Biological Activity of *Illicium verum* (Star Anise) on *Lernaea cyprinacea*-Infested *Carassius auratus* (Goldfish): In Vivo Study

**DOI:** 10.3390/life12122054

**Published:** 2022-12-07

**Authors:** Marwa M. Attia, Amal M. Alzahrani, Magdy I. Hanna, Heba M. Salem, Mohammed A. S. Abourehab, Mohamed T. El-Saadony, Hasnaa Thabit

**Affiliations:** 1 Department of Parasitology, Faculty of Veterinary Medicine, Cairo University, Giza 12211, Egypt; 2 Department of Biology, Faculty of Arts and Science in Almandaq, Al Baha University, Al Baha 65799, Saudi Arabia; 3 Department of Aquatic Animal Medicine and Management, Faculty of Veterinary Medicine, Cairo University, Giza 12211, Egypt; 4 Department of Poultry Diseases, Faculty of Veterinary Medicine, Cairo University, Giza 12211, Egypt; 5 Department of Pharmaceutics, College of Pharmacy, Umm Al-Qura University, Makkah 21955, Saudi Arabia; 6 Department of Agricultural Microbiology, Faculty of Agriculture, Zagazig University, Zagazig 44511, Egypt; 7 Department of Zoology and Entomology, Faculty of Science, Assiut University, Assiut 71515, Egypt

**Keywords:** goldfish, gene expression, *L. cyprinacea*, *Illicium verum*, *q*PCR, scanning electron microscope

## Abstract

*Lernaea cyprinacea* infestation is considered a serious economic problem in the fish market. An assessment to control this parasite is needed to manage this problem. The *Illicium verum* oil extract has considerable antioxidant activity and scavenges 96.22% of free radicals; the high antioxidant activity refers to the phenolic content presence. The extract contains minerals, especially K, fibers, and dry matter. So, the *Illicium verum* ingredients were tested against this copepod for in vitro and in vivo investigation with the assessment of the treatment trial using a scanning electron microscope and evaluating the change in different immunological genes in goldfish. Female parasitic *L. cyprinacea* worms were blackish and hairy. The in vitro study on *L. cyprinacea* adults using star anise revealed that the LC50 was 12.5 and 25 μg/mL for 2 and 1 h exposure periods, respectively. Interleukin (IL-1β) and IL-6 were grossly upregulated in *C. auratus-infested* skin by *L. cyprinacea* after treatment by 1 week, then declined after 3 weeks. In contrast, TNF-α was 18 folds upregulated in the first week after treatment, with a decline after 3 weeks. In conclusion, star anise is recommended as a safe and economical agent for controlling *L. cyprinacea* infestation in fish.

## 1. Introduction

*Lernaea cyprinacea*, popular as the anchor worm, is a lernaeid copepod. The most dangerous parasite can infect freshwater fish, particularly juvenile ones [1,2]. The adult parasite enters the body, then becomes deeply implanted in the tissues of the fish host [3]. With global distribution, this species (spp.) is considered a serious invasive external parasite on fish. *Lernaea cyprinacea* is widely spread in freshwater crustaceans and is accompanied by different water hosts such as tadpoles and fish [4,5].

The Cyprinidae family is the most susceptible fish family, involving common carp, goldfish, and koi [6]. Only adult female worms infect the fish fins, gills, integument, eyes, nostrils, and oral cavity, but the preferable site is the base of the fins and abdominal region [7]. Their mouths and two pairs of anchors are inserted into the host muscles, and they have an extended thready body with two trailing egg sacs.

Although mass fish deaths from *L. cyprinacea* infestations are rare, repeated infestations and prolonged contact can reduce fish development and productivity and negatively damage their looks and health [8,9]. Individual parasites can produce hemorrhagic and ulcerated lesions and severe localized damage to the affected tissue. Secondary bacterial and fungal infections probably occur because these lesions can lead to death [1]. The mechanical disconnecting of the female parasite on a large scale is problematic since the removal procedure is often partial, leaving the anchor within the fish cells.

The most-often employed insecticides against *L. cyprinacea* are potassium permanganate and organophosphate pesticides. However, they typically influence the stages of the parasites that survive on the host more than adult worms, and safety measures versus potential fish toxicity must be taken [1]. To guarantee that many chemical therapy protocols destroy all parasitic stages against this copepod, the recommended dose for chemotherapeutic agents is once a week, repeated four times [1]. 

*Lernaea cyprinacea* copepods may be easily destroyed by bathing in organophosphate solutions [10,11,12]. Usually, organophosphate or synthetic pyrethroid solutions are used to get rid of ectoparasites [10]. Medicinal herbs and their derivatives are increasingly used in aquaculture worldwide because they are biodegradable, easy to find and grow, and do not build up in animal tissues [13].

Various materials were used as phyto-therapeutic agents in sustainable aquaculture to overcome parasitic copepod infestations [14]. Terpenes, terpenoids, phenyl-propenes, and isothiocyanates are the main active groups in EOs that are considered a potent biocidal for bacteria and parasites. EOs are made up of various chemicals that do not have a unique cellular target in parasites [15]. The essential oils’ (EOs)—monoterpenes-pinene and sabinene—have been shown to have antiparasitic action. Furthermore, the synergistic impacts of multiple mixtures in EOs are another important property demonstrating a greater mode of action than single drugs. Because EOs are hydrophobic and can get into cells, potassium ions and cytoplasmic components leak out of parasitic cells, altering the cells’ shape and stopping the parasites from completing their job [16].

Ding et al. [17,18] and Patra et al. [19] found that anethole was the main compound in star anise (85–90%), followed by estragole, and anisaldehyde. It is worth noting that star anise and its extracts have a lot of the aromatic plant’s flavor [20]. However, this strategy has not been used due to negative experiences with pesticide toxicity. It is unclear whether *Illicium verum* has biocidal properties against *L. cyprinacea*. A novel strategy to overcome *L. cyprinacea* utilizing this plant extract was tested. Before and after treatment, different cytokines released during the infection were studied using a scanning electron microscope (SEM) and immunological assessment. Lastly, the antiparasitic effects of *Illicium verum* on *L. cyprinacea* were examined, both in a test tube and a living animal.

## 2. Materials and Methods

### 2.1. Fish Inspection and Samples’ Collection

Anchor worms that had infested a local goldfish merchant in Egypt’s Giza governorate in September 2021 were collected. The history, manifestations, incidence, and intensity of the illness were all reported during the investigation. One hundred and twenty contaminated adult Goldfish (*Carassius auratus*) were caught and delivered to the Faculty of Veterinary Medicine, Cairo University’s parasitology lab, in a 50-L tank, accompanied by aeration. The goldfish ranged from 9–15 cm in length and from 80–120 gm in weight. Adult and surviving copepods were picked up by a mechanical method from the fish, identified, and described in the lab [21]. All institutional guidelines for the animals’ care and handling were considered. The Ethical Committee approved this study of animal use and their care under registration number: 17300826.

### 2.2. Collection of the Plant Extract

**Figure 1:**
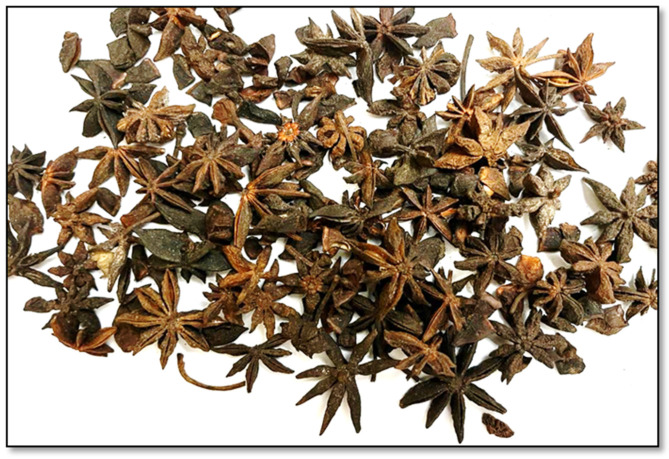
The star anise (*Illicium verum*) used in this study.

*Illicium verum* L. (Figure 1) was dried and powdered in an oven at 40 °C. Maceration with 250 mL of methanol or dichloromethane for three hours at room temperature with shaking was used to extract the dried powdered material (50 g). Through the use of hydro-distillation, anise oil was extracted and filtered. Then, 100 g of dried star anise fruit were crushed into bits and extracted with 800 mL of deionized water in a round bottom flask with a Clevenger-style setup under 70 °C for 24 h, as reported by Politeo et al. [22]. Filtering was executed on the extracts. Then, the majority of the water was removed. The addition of anhydrous MgSO_4_ dried the extracts from anise oil. The suspension was produced as pure anise oil after settling [19,23].

### 2.3. Chemical Composition of Illicium verum Extract

The oil extract of *Illicium verum* was dissolved in Dimethyl sulfoxide (DMSO) for the following examination. Moisture was determined by drying in an air oven at 110 °C until constant weight; crude protein was evaluated using the Micro-Kjeldahl method to determine the total nitrogen and multiplying its value by 6.25. The oil content was evaluated in a Soxhlet apparatus using *n*-hexane (40–60 °C) as a solvent; ash content was determined by ashing in an electric muffle at 550 °C until a constant weight. The crude fiber content was calculated using the technique described [24]. All determinations were made using the indicated procedures [25]. Carbohydrates were determined using the difference technique (=100 − (% protein + % fat + % ash + % fiber) [26]. The wet ashing technique was used to assess mineral concentrations [25]. Na, K, Mn, Co, Zn, Fe, Cu, Ni, and P were assessed at the Central Laboratory, Kafeel Sheikh University, utilizing the atomic absorption (NC.9423-400-30042) England method, as outlined by AOAC [25].

Singleton et al. [27] measured the total soluble phenolics of the DMSO fraction using the Folin–Ciocalteu reagent and gallic acid as the standard phenolic compound [28]. In separate test tubes, 40 µL of DMSO fraction and standard were transferred, and 200 µL of diluted Folin–Ciocalteu reagent was added. The mixture was stirred thoroughly, left for eight minutes, and then added to 600 µL of sodium carbonate solution 7.5% while stirring continuously. A spectrophotometer measured the solution’s absorbance at 750 nm after incubation for one hour in the dark. *Illicium verum* oil extract’s total phenolic components were measured in mg of the gallic acid standard curve (G.A.E.) [29,30].

Kim et al. [31] evaluated the total flavonoid content of the DMSO fraction using catechin as a reference. A total of 1 mL of DMSO fraction (100 g/mL) was mixed with 4 mL of purified water and 300 µL of aluminum chloride. Five minutes were spent incubating the mixture at room temperature. At 510 nm, the absorbance of samples was measured. Regarding catechin equivalents (mg/g), the total flavonoid content of the DMSO fraction was expressed [32].

The chemical composition of star anise extract is presented in Table 1. The extract has considerable antioxidant activity and scavenges 96.22% of free radicals; the high antioxidant activity refers to the phenolic content presence. The extract contains minerals, especially K, fibers, and dry matter.

### 2.4. HPLC Phenolic Profiling of Star Anise Extract

The HPLC Shimadzu series (Shimadzu-prominence-20A, Tokyo; Japan) was utilized to identify the phenolic profile in the star anise extract. The mobile phase was 0.01% acetic acid in water (A) and acetonitrile. The stationary phase was a separation column (Gemini, C18) with a 2 mL/min flow rate (4.6 × 150 × 5 um) and a 2 mL/min flow rate (4.6 × 150 × 5 um) (B). Monitoring and controlling the HPLC pumps, autosampler, column, oven, and diode array system. Class VP software was used to analyze the chromatographic results (Shimadzu 5.0) [33,34,35]. The phenolic compounds were analyzed at 280 nm and the flavonoids at 370 nm.

Table 2 shows the phenolic profile of star anise extract by HPLC, where Vanillic (5704.35), Protocatechuic (6052.15), Coumarin (18,384.35), Chlorogenic (11,932.1), Iso ferulic (5592.05), Luteolin 7 glucose (5151.4), Apigenin 6-rhamnose 8-glucose (5333.2505), and Apigenin-7-0-neohes (15271 mg/L) were found in high content, while medium contents of Ferulic, (4071.41), P-OH benzoic, (2148.85), Caffeine, (4094.1), Catechol, (2501), Caffeic, (2711.5), Cinnamic, (4457.35), Benzoic, (4575.5), 4-amino benzoic acid, (4228.05), Salicylic, (4873.23), Naringin, (4424.44), Acacetin neo. rutinoside, (3802.675), Quercetin, (3753.705), and Quercitrin, (2427.615) were found in the star anise extract. These phenolics protect against the parasite and oxidative stresses [36].

### 2.5. Volatile Compounds in Star Anise Essential Oil by GC-Mass

For determination of the chemical composition of basil leaves, leaves of peppermint, and clove buds EOs, gas chromatography–mass spectrometry (GC-MS) analysis was conducted using GC-2010 Shimadzu capillary gas chromatography directly coupled to the mass spectrometer system (GC-MS–model QP 2010; (Shimadzu, Kyoto, Japan)) DB-c18 column under the following conditions: The injector temperature was 250 C. Oven temperature program: 30 °C for 2.0 min, then ramp to 250 °C at a rate of 2.0 degrees Celsius per minute for 5.0 min. The MS source temperature was 200 °C, electron energy was 70 eV, the carrier gas was helium at a flow rate of 1.4 mL/min, and 1 µL of each diluted sample in n-hexane (1:1, *v*/*v*) was injected. EI spectra were scanned from 43.00 to 600 *m*/*z* to identify peaks through NIST mass data-search libraries and the highest REV and similarity indicators’ hits. The sample components were identified by comparing their relative indices and mass spectra with the computer controlling the GC-MS system [37,38].

Table 3 shows the VOCs in star anise essential oil. The main compound in the essential oil is (E)-Anethole* accounted for 89.24% of oil, followed by lower contents. Volatile organic compounds exhibited powerful potential against parasites [39].

### 2.6. LC50 of Illicium verum on L. cyprinacea

*Illicium verum* was evaluated for testing the mortality of *L. cyprinacea,* which was diluted with phosphate-buffered saline, making two-fold serial dilutions of (25 μg/mL, 12.5 μg/mL, 6.25 μg/mL, 3 μg/mL, 2 μg/mL, 1 μg/mL) [40,41]. Commercial clove oils (Ectyo-clove^®^; Paris, France) were used to anesthetize *L. cyprinacea*-infested fish to remove the live female parasite manually. Two hundred adult surviving parasite females of *L. cyprinacea* were harvested and separated into 20 *L. cyprinacea*/dilution/replicate groups. The copepods were inspected using a light microscope after exposure to graded *Illicium verum* dilutions for 1, 2, 4 and 8-h periods, and the mortality rate was recorded [21].

### 2.7. Demonstration of the Effect of the Illicium verum on L. cyprinacea by SEM

Five parasite females of *L. cyprinacea* subjected to *Illicium verum* were harvested and rinsed multiple times with saline. The copepods were rinsed multiple times with saline, then cleaned in lactophenol and loaded on gelatin to analyze their morphology [21,42]. Five freshly obtained copepods were rinsed multiple times in saline. Adult *L. cyprinacea* was immersed in 2.5% *glutaraldehyde.* After that, the specimens were dehydrated in an increasing ethanol series, dried in a CO_2_ critical point drier (Autosamdri-815; Lewis Avenue, Rockville, ML, USA), bonded over stubs, and sputter-covered with twenty nm gold (Spi-Module sputter coater; UK). Then, the specimens were inspected and photographed using a scanning electron microscope (SEM) at magnifications ranging from 35 to 500 (JEOL JSM 5200 Electron Probe Microanalyzer; Tokyo, Japan) [21].

### 2.8. In Vivo Parasiticidal Efficacy of the Illicium verum

In glass aquariums of 20 cm × 30 cm × 40 cm, fifteen adult goldfish infected with adult *L. cyprinacea* were placed in 10 L of tap water after chlorine removal. They were kept in the lab for three days before being exposed. The fish were exposed to 12.5 μg/mL of *Illicium verum* for 2 h. The water was then drained and replenished with new water. The fish were checked for 14 days post-infection and were supplied with commercial fish pellet feed once per day at 1% body weight.

### 2.9. Evaluation of Biological Parameters

The skin was dissected from the fish infested with copepods and control non-infested fish groups (healthy fish) and the treated fish 1 and 3 weeks after exposure to star anise; all samples were kept aseptically at −20 °C for subsequent research.

### 2.10. Extraction of mRNA

According to the manufacturer’s instructions, a Total RNA kit (Ambion; Applied Biosystems) was used to extract mRNA from 100 mg of the integument. Using a FastPrep-24 homogenizer (MP Biomedicals; Bio Laboratories Pte Ltd Lobby A, Ubi Techpark, Singapore) 2 cycles of 30 s at 6 m/s, the fish’s skin was homogenized and placed in Lysing Matrix D tubes (MP Biomedicals). The quantity and purity of the RNA were determined by Nanodrop (Thermo Scientific, Waltham, MA, USA). According to the manufacturer’s recommendations, 500 ng of mRNA were obtained using DNaseI amplification grade (Invitrogen). The reverse transcription of treated RNA was achieved following the approach described by Tu et al. [43] and Younis et al. [44], and the High-Capacity cDNA Archive Kit (Applied Biosystems) was used. Table 4 lists the *q*RT-PCR primer sets specific for TNF-α, IL-1β, and IL-6 specific for *Carassius auratus* based on the sequences deposited in the GenBank; samples were obtained from 1 cm of skin and muscles infested with *L. cyperinacea*.

The approach described by Attia et al. [45,46,47] was followed for the extraction and synthesis of the analyzed mRNA. In a Step One™ Real-Time PCR System (Applied Biosystems, Bedford, MA, USA). 10 L of (SYBR^®^ Premix Ex Taq TM (Tli RNase H Plus), ROX Plus (Takada 3-chome, Toshima-ku, Tokyo; Japan), 1 L of cDNA, and 0.5 L of the produced primer (100 nM) were mixed with 20 L of ultra-pure water.

The used cycling conditions were followed by Attia et al. [45]. The ^∆^CT value was determined by subtracting the controlled gene (β-actin), ∆ CT, as an internal control, from the result of the examined gene. Conditions for PCR cycling in a 40-cycle amplification, denaturation for 30 s at 94 °C, annealing for 30 s at 60 °C, and extension for 45 s at 72 °C were used for the 40-cycle amplification. Tu et al. [43] developed a real-time PCR technique. Three times the samples were taken.

### 2.11. Determination of Cytokines

TNF- (ng/mL), IL-1 (pg/mL), and MCP-1 (ng/mL) cytokine levels were measured in serum samples using the enzyme-linked immunosorbent assay (ELISA). The Human IL-1β Bioassay Technology Laboratory (BT-Lab; Shanghai, China) ELISA kit (Cat. No. E0143Hu), Human TNF-ELISA kit (Cat. No. E0082Hu), and Human MCP-1 ELISA kit (Cat. No. E0124Hu) ELISA procedures were followed. The ELISA procedure was carried out by adding 40 μL of a serum sample to the sample well. After that, it added 50 μL of streptavidin-HRP to the sample wells and standard wells. The wells were covered and incubated for 60 min at 37 °C, then washed with Wash Buffer five times. First, 50 μL of substrate solution, and then each well was attached, and 50 µL of substrate solution B was added. Next, it was incubated in the dark for 10 min at 37 °C. Following that, 50 µL of Stop Solution was added to each well. Within 10 min of adding the stop solution, each optical density (OD) value was correctly read with a microplate reader at a wavelength of 450 nm.

### 2.12. Total Protein Evaluation in Fish

Total protein was determined using Coomassie Brilliant Blue (G-250) [48]. Briefly, a solution of Coomassie Brilliant Blue dissolved in 95% ethanol was prepared at a final concentration of 20 mg/mL. Phosphoric acid (85%) was added in a ratio of 1:2, the mixture was stirred till the addition of water to a final concentration of (15%, *v*/*v*). The filtered solution was kept at 4 °C. A total of 100 mL fish filtrate sample was mixed with 5 mL Bradford reagent for 5 min. Bovine Serum Albumin was used to construct a standard curve for the quantification of total protein in samples (y = 0.0009x + 0.0148). The absorbance was measured at 595 nm [49].

### 2.13. Statistical Evaluation

SPSS version 21 was applied to analyze the mean of the triplicate data. Kirthi et al. [50] used probit analysis to determine the 50% mortality concentration with 95% confidence intervals. The LC50 was determined using the obtained regression equation (Y = mortality percent; X = log concentration) or by drawing a transverse line passing from probit 0.5 on the y-axis to the x-axis and determining the related log; concentration was then inversed to assess the LC50.

## 3. Results

**Figure 2:**
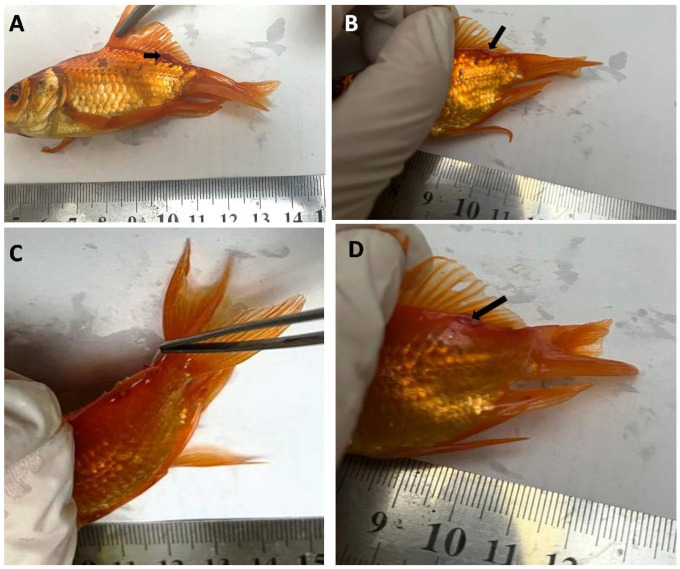
The infested goldfish with *L. cyprinacea* appear as hairs attached to the fins of the fish (**A**–**D**) (referred to with arrows). (**A**,**B**): Infested goldfish with *L. cyprinacea* on fins, referred to with an arrow; (**C**): The infestation with *L. cyprinacea* appeared as thread-like and was attached with forceps; (**D**): The site of the *L. cyprinacea* infestation was surrounded by an ulcer and hemorrhagic area, referred to with an arrow.

Recurrent infections have been reported in adult goldfish and fingerlings, especially in the spring and autumn. Female parasitic *L. cyprinacea* seemed to be blackish hairy worms involved in focal locations on the host’s flanks, flank area, beneath the operculum, dorsal, and tail fins with the presence of ulcers around the attachment of the copepods (Figure 2).

**Figure 3:**
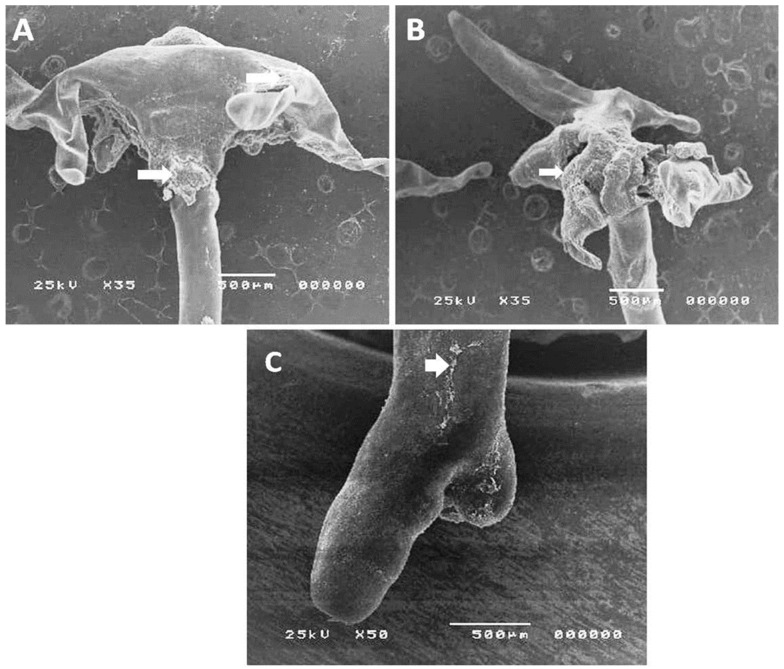
Scanning electron microscopic study of *L. cyprinacea* infesting goldfish aquaria after treatment with star anise (*Illicium verum*). (**A**,**B**): anchor of the copepod showing severe corrugation and shrinkage which referred by arrows. (**C**): posterior end of the copepod showing the destruction of the cuticle of the copepod; referred by arrows.

**Figure 4:**
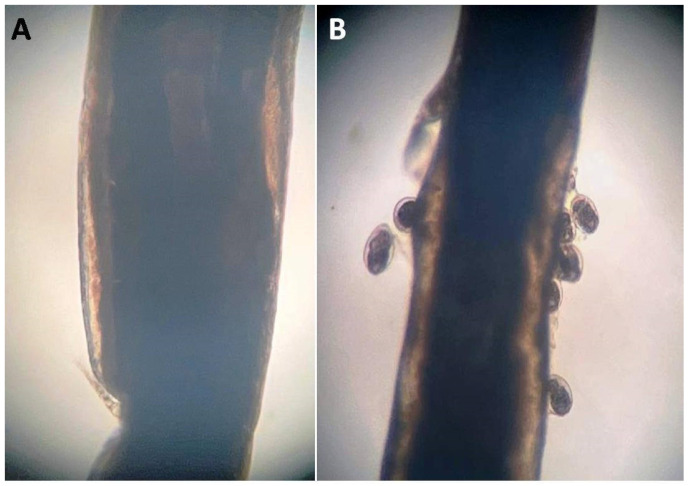
Light micrograph study of *L. cyprinacea* infesting goldfish aquaria after treatment with star anise (*Illicium verum*); showing destruction of the cuticle. (**A**): normal cuticle; (**B**): destructed cuticle with release of the eggs.

The LC50 of *Illicium verum* on *L*. cyprinacea is recorded in Table 5. The recorded LC50 was 12.5 μg/mL for 2 h, and 25 μg/mL for a one-hour exposure period. SEM analysis of *L. cyprinacea* treated with *Illicium verum* revealed that the anchor was corrugated and shrinkage showing wrinkles, longitudinal folds, and a malformed body with a severely corrugated and reduced posterior end (Figure 3 and Figure 4).

**Figure 5:**
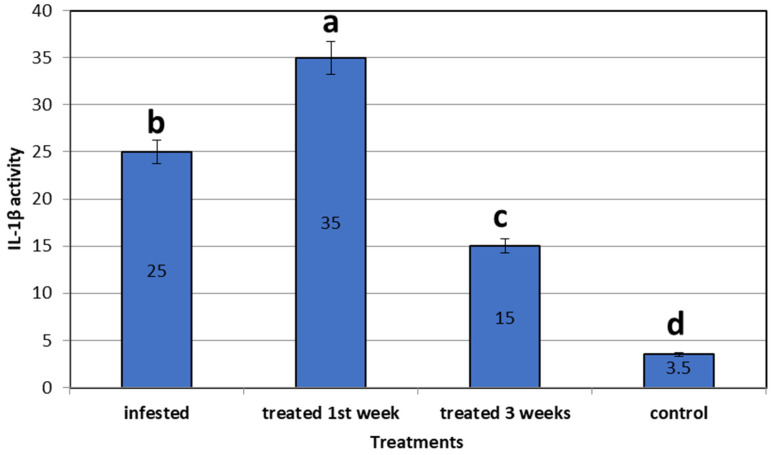
Gene expression analysis (IL-1β) of mRNA of goldfish infested with *L. cyprinacea* before and after treatment with *Illicium verum* (quantitative real time PCR analysis of immunological gene of interleukin1 β in goldfish before and after treatment trial). Different lowercase letters above columns indicates significant differences. Columns legend; Goldfish (control not infected or added *Illicium verum*), *Goldfish* Infested with *L. cyprinacea,* Goldfish treated with *Illicium verum* for 1 week, *Goldfish* treated with *Illicium verum* for 3 weeks.

The female *L. cyprinacea* had completely separated from the afflicted fish after 2–7 days of exposure to *I. verum*. The remaining lesions looked to have healed 7 days post-exposure. The treated fish appeared healthy during the monitoring period, with no signs of re-infestation. In the *C. auratus* skin infested by *L. cyprinacea*, IL-1β was 25-fold more upregulated than in the control non-infested fish. Concerning the expression of mRNA of the treated fish using star anise, IL-1β was 35-fold upregulated in the first week after treatment, then declined after 3 weeks to 15-fold more than in the control non-infested fish. All genes were expressed significantly higher than the control non-infested group (3.50 ± 0.00) (*p* = 0.0001) (Figure 5).

**Figure 6:**
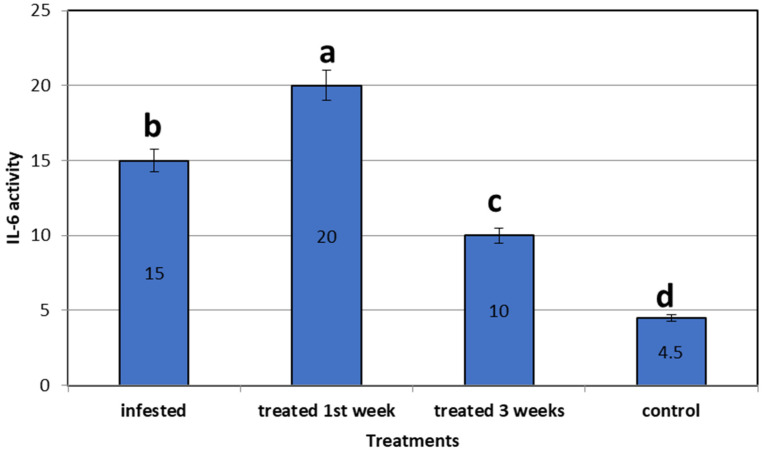
Gene expression analysis (IL-6) of mRNA of goldfish infested with *L. cyprinacea* before and after treatment with *Illicium verum* (quantitative real time PCR analysis of immunological gene of interleukin 6 in goldfish before and after treatment trial). Different lowercase letters above columns indicate significant differences. Columns legend; *Goldfish* (control not infected or added *Illicium verum*), *Goldfish* Infested with *L. cyprinacea, Goldfish* treated with *Illicium verum* for 1 week, *Goldfish* treated with *Illicium verum* for 3 weeks.

In the *C. auratus* skin infested by *L. cyprinacea*, IL-6 was 15-fold more upregulated than in the control non-infested fish. Concerning the expression of mRNA of the treated fish using star anise, IL-6 was 20-fold upregulated in the first week after treatment, then declined after 3 weeks of treatment to 10-fold more than in the control non-infested fish. All genes were expressed significantly higher than the control non-infested group (4.20 ± 0.00) (*p* = 0.0001) (Figure 6).

**Figure 7:**
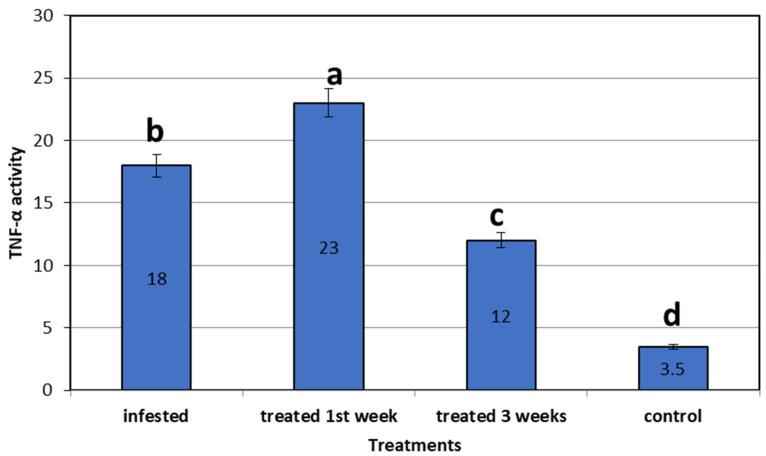
Gene expression analysis TNF-α of mRNA of goldfish infested with *L. cyprinacea* before and after treatment with *Illicium verum* (quantitative real time PCR analysis of immunological gene of TNF-α in goldfish before and after treatment trial). Different lowercase letters above columns indicate significant differences. Columns legend; *Goldfish* (control not infected or added *Illicium verum*), *Goldfish* Infested with *L. cyprinacea, Goldfish* treated with *Illicium verum* for 1 week, *Goldfish* treated with *Illicium verum* for 3 weeks.

In the *C. auratus* skin infested by *L. cyprinacea*, TNF-α was 18-fold more upregulated than in the control non-infested fish. Concerning the expression of mRNA of the treated fish using star anise, TNF- α was 23-fold upregulated in the first week after treatment, then declined after 3 weeks of treatment to 12-fold more than in control non-infested fish. All genes were expressed significantly higher than the control non-infested group (3.50 ± 0.00) (*p* = 0.0001) (Figure 7).

**Figure 8:**
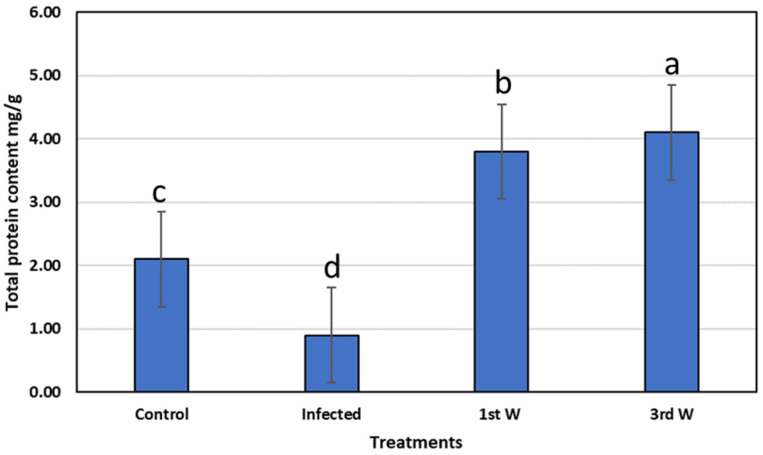
Total protein content in goldfish before and after treatment with *Illicium verum*. Different lowercase letters above columns indicate significant differences. Columns legend; *Goldfish* (control not infected or added *Illicium verum*), *Goldfish* Infested with *L. cyprinacea, Goldfish* treated with *Illicium verum* for 1 week, *Goldfish* treated with *Illicium verum* for 3 weeks.

At the level of protein expressed in fish after treatment with star anise extract, the total protein content decreased in infected fish, i.e., 0.9 mg/g as compared to the control 2.1 mg/g; after one-week treatment with star anise extract in the fish diet the total protein increased with a relative increase of 80 and 322% compared to control and infected fish; (Figure 8).

## 4. Discussion

*Illicium verum* (Magnoliaceae) is found throughout Asia’s tropical and subtropical zones. The fruits are widely available in the marketplaces. Star anise is a popular spice. The oil is used as a flavoring, and the spice is used for flavoring food [17]. Star anise oil has tonic, appetite stimulant, carminative, diuretic, and moderate expectorant properties [51]. It is a popular element in cough treatments and is useful for flatulence, spasmodic discomfort, and colic. Topically, the oil is used to treat rheumatism and otalgia and as an antibacterial [51].

Lernaeosis is a disease caused by copepod parasites of the genus *Lernaea* (Linnaeus, 1758) (Cyclopoida: Lernaeidae), which can infest a variety of freshwater fish spp., having major pathogenic consequences on their hosts [52,53]. *L. cyprinacea* causes critical issues in the marketing of ornamental fish. The current investigation detected a severe *L. cyprinacea* infection in a goldfish glass aquarium; the female worms were connected to the integument of the flank area and fin base, as these parts provide high defense versus the water currents [7,54].

They can cause bleeding and necrosis on the gills, resulting in a reduction in breathing capacity. The adult worm also infects the fish, attaching itself deeply in the cells and revealing lesions to the tegument, external muscular cell, eye, and, in some circumstances, even the internal systems. Young fishes are particularly vulnerable, as even a few parasites can kill them [55,56].

Different control measures of the disease include chemical, biological, plant, or natural control agents. Among them, the natural one is the best control. It is a new alternative method for sustainable aquaculture using EOs or other herbal items to combat microbial agents and parasitic illnesses [52,57]. 

In this study, star anise was evaluated for antiparasitic activity in vitro and in vivo against *L. cyprinacea*, with a good result in low concentration where the LC50 was 12.5 μg/mL for a 2 h exposure period; or for 25 μg/mL for 1 h exposure. This study uses direct contact and fumigation approaches such as the previous study on arthropods (Drosophila melanogaster) with mortality using star anise in vitro. The main component of the methanol extract was (E)-anethole. The 5-day LD50 of (E)-anethole in *D. melanogaster* was a diet containing 0.2 mg/mL [20,58]. In addition, the insecticidal effect of (E)-anethole was demonstrated versus adult *Blattella germanica* [40] at 0.159 mg/cm2 one and three days post-treatment. (E)-anethole induced an 80.3 percent mortality in a filter paper diffusion approach, whereas hydramethylnon (positive control) caused 93.3 percent mortality.

Essential oil from the fruit of *I. verum* was also found to have insecticidal activities against *Callosobruchus chinensis*, *Botrytis cinerea*, and *Colletotrichum gloeosporioides* larvae and adults [59,60,61]. *I. verum* oil had an LC50 of 12.5 and 11.1 μL against *C. chinensis* adults and larvae. The LC50 for *T. castaneum* adults and larvae was 19.87 and 18.43 μL, respectively. Maini and Morallo-Rejesus [62] investigated the toxicity of star anise oil to golden snails, finding that 10–20 ppm caused 100 % mortality in juvenile snails. Meanwhile the previous study of cat flea control using star anise was recorded by Freitas et al. [41], who recorded 100% mortality when using 400 μg.cm^2^ after a 48-h exposure period.

Our results revealed that the proinflammatory cytokines (IL-1β, IL-6, and TNF-α) were increased during the infestation with *L. cyprinacea* in goldfish; these findings are similar to those recorded by Tu et al. [43], which applied to goldfish infested with *Gyrodactylus kobayashii* and the cytokines were again elevated during treatment with the star anise. These results are supported by those recorded by Vaseeharan and Thaya [63]. They stated that essential oils are also immunostimulants. Zhang et al. [64] and Sarhadi et al. [65] stated that feeding *Cyprinus carpio* with *Origanum vulgare* and *Artemisia annua* for eight weeks improved both the immunological and antioxidant qualities, which increased IL-1β, as well as resistance to *A. hydrophila*. EOs boosted IL-1β and IL-10 transcription while decreasing TNF-α and transforming growth factor (TGF-β).

The interleukin-6 (IL-6) family of cytokines performs various biological functions and exhibits a high level of redundancy within the family. Although several mRNA transcripts from teleost fish resembling cytokines of the IL-6 family have been discovered, none have been functionally characterized to establish their identity. We describe the discovery and functional analysis of goldfish M17, a molecule with sequence and mRNA expression patterns resembling ciliary neurotrophic factor and mammalian leukemia inhibitory factor in mammals and birds.

Besides the beneficial uses of star anise in therapy for different diseases, there are several case reports relating to juveniles recording neurotoxicity and hepatotoxicity, such as those recorded in [66,67,68]. However, those investigations were in juveniles and employed homemade herbal remedies; though several investigations and extensive research were completed in relation to this plant to prove these case reports. In addition, this plant gave good results in relation to different parasites.

Essential oil-based biopesticides have a bright future [66,67,68]. EOs of star anise are highly effective, have multiple modes of action (suitable for usage versus insect resistance), and are low in toxicity involving people. Additionally, obtaining EOs is often simple and inexpensive, and the risk of intoxication is negligible [67].

## 5. Conclusions

In conclusion, for the first time, the anti-copepodal effect and residual value of the EOs of star anise were detected in this study. The pharmaceutical and agronomy industries are interested in developing biopesticides for veterinary and fisheries’ medication, since the star anise EOs are safe and environmentally friendly and can be used against parasites infesting aquatic organisms.

## Figures and Tables

**Table 1:** Chemical composition of star anise (*Illicium verum*).

Chemical Composition	Content (mg/L)
Approximate analysis	
Protein	6.5 ± 0.1
Fat	3.76 ± 0.2
Fiber	28.12 ± 0.5
Carbohydrates	59.33 ± 0.6
Ash	4.08 ± 0.1
Dry matter	86.65 ± 0.3
Antioxidant content	
Total phenols (mg/L)	33.36 ± 0.9
Total flavonoids (mg/L)	15.66 ± 0.2
Antioxidant activity (%)	96.22 ± 1.1
Minerals (mg/L)	
Zn	14.2 ± 0.2
Fe	58.3 ± 0.1
Cu	15.3 ± 0.3
Mn	115.3 ± 1.3
Co	ND
Ni	ND
P	912.30 ± 1.8
K	5651.96 ± 1.1
Na	860.61 ± 0.9

Data are presented mean ± SD.

**Table 2:** Phenolic compounds of star anise (mg/L).

Phenolic Compound	Concentration (mg/L)
Vanillic	5704.35 ± 0.1
3.4.5. methoxy cinnamic	1448.9 ± 0.9
Catechin	2525.8 ± 0.5
Protocatechuic	6052.15 ± 0.1
Ferulic	4071.41 ± 0.6
Coumarin	18,384.35 ± 0.1
P-OH benzoic	2148.85 ± 0.6
Gallic	1750.3 ± 0.8
Caffeine	4094.1 ± 0.4
P-Coumaric	325.8 ± 0.7
Catechol	2501 ± 0.3
Caffeic	2711.5 ± 0.5
Cinnamic	4457.35 ± 0.3
Chlorogenic	11,932.1 ± 0.8
Iso ferulic	5592.05 ± 0.9
Benzoic	4575.5 ± 0.7
4-amino benzoic acid	4228.05 ± 0.6
alpha Coumaric	6583.44 ± 0.1
Salicylic	4873.23 ± 0.3
Rutin	1524.685 ± 0.8
Naringin	4424.44 ± 0.3
Apigenin	531.975 ± 0.2
Naringenin	1097.1415 ± 0.1
Acacetin neo. rutinoside	3802.675 ± 0.3
Luteolin 7 glucose	5151.4 ± 0.1
Apigenin 6-rhamose 8-glucose	5333.25 ± 0.9
Apigenin 6-arabinose 8-glactose	1473.28 ± 0.7
Hesperetin	588.35 ± 0.8
Kaempferol	1277.9 ± 0.3
Quercetin	3753.705 ± 0.5
Quercitrin	2427.615 ± 0.6
Apigenin-7-0-neohes	15,271 ± 0.4
Kaempferol 3-2-p-coumaroylglucose	3578.7 ± 0.1

Data are presented mean ± SD.

**Table 3:** Volatile compounds profile in star anise essential oil.

Rt	Volatile Compounds	Area%
2.19	Furfural	0.01 ± 0.001
3.24	α-Thujene	0.01 ± 0.0002
3.34	α-Pinene	0.26 ± 0.005
3.63	Camphene	0.01 ± 0.00
4.12	β-Pinene	0.03 ± 0.01
4.40	Myrcene	0.12 ± 0.08
4.67	α-Phellandrene	0.51 ± 0.007
4.86	α-Terpinene	0.08 ± 0.004
5.10	Limonene	2.60 ± 0.1
5.15	1,8-Cineole	0.15 ± 0.02
5.27	*cis*-β-Ocimene	0.01 ± 0.001
5.45	*trans*-β-Ocimene	0.05 ± 0.002
5.64	γ-Terpinene	0.11 ± 0.004
5.94	*cis*-Linalool oxide (fur.)	0.01 ± 0.009
6.11	Terpinolene	0.12 ± 0.001
6.25	*trans*-Linalool oxide (fur.)	0.05 ± 0.006
6.33	para-Cymene	0.02 ± 0.001
6.64	Linalool	0.61 ± 0.002
7.98	para-Vinyl anisole	0.01 ± 0.007
8.68	Terpinen-4-ol	0.19 ± 0.003
9.37	α-Terpineol	2.18 ± 0.2
11.63	(*Z*)-Anethole	0.28 ± 0.004
11.85	Geraniol	0.02 ± 0.001
12.30	para-Anisaldehyde	0.30 ± 0.002
14.00	(*E*)-Anethole	89.24 ± 0.1
17.40	α-Copaene	0.08 ± 0.002
19.10	para-Methyl anisate	0.04 ± 0.003
19.40	Geranyl acetate	0.05 ± 0.002
19.85	para-Acetonyl anisole	0.12 ± 0.001
20.24	*cis*-α-Bergamotene	0.41 ± 0.004
21.78	*trans*-α-Bergamotene	0.33 ± 0.02
22.99	α-Humulene	0.02 ± 0.001
25.98	Viridiflorene	0.03 ± 0.001
28.40	γ-Cadinene	0.10 ± 0.02
28.87	δ-Cadinene	0.03 ± 0.001
29.60	Methyl (E)-isoeugenol	0.03 ± 0.005
33.66	(E)-Nerolidol	0.07 ± 0.004
34.08	Globulol	0.02 ± 0.002
35.85	para-Methoxyphenyl-1,2-	0.02 ± 0.001
	propanediol epimer	
37.91	τ-Cadinol	0.30 ± 0.01
39.24	(Z)-Foeniculin	0.79 ± 0.02

Rt: Retention time; Data are presented mean ± SD.

**Table 4:** Primers used in the Gene expression analysis of the goldfish (*Carassius auratus).*

Genes	Sequence (5′->3′)	Accession No.
IL-1β	F: GATGCGCTGCTCAGCTTCT	AJ249137
	R: AGTGGGTGCTACATTAACCATACG	
IL-6	F-CTGGCCAGACCATATCGCAG	DQ861993
	R-TTCTGTTCTTGAACTGCTTGACT	
TNF-α	F: CATTCCTACGGATGGCATTTACTT	EU069817
	R: CCTCAGGAATGTCAGTCTTGCAT	
β-Actin	F: GATGCGGAAACTGGAAAGGG	AB039726
	R: ATGAGGGCAGAGTGGTAGACG	

**Table 5:** The anti-copepodal activity of *Illicium verum* on *L. cyprinacea* (Preliminary screening).

Tested Concentration	*L. cyprinacea* Mortality				
	M.M. % ± S.E				
	15 min	1 h	2 h	4 h	8 h
25 μg/mL	20.9 ± 0.56	50 ± 0.55	30.0 ± 0.00	00 ± 0.00	00 ± 0.00
12.5 μg/mL	10 ± 0.56	40.00 ± 0.94	50 ± 0.50	00 ± 0.00	00 ± 0.00
6.25 μg/mL	05 ± 0.36	20 ± 0.48	40 ± 0.47	30 ± 0.77	05 ± 0.50
3 μg/mL	00 ± 0.00	10.0 ± 0.76	20.0 ± 0.59	25.0 ± 0.50	30.0 ± 0.57
2 μg/mL	00 ± 0.00	00 ± 0.00	00 ± 0.00	20.5 ± 0.36	30.98 ± 1.50
1 μg/mL	00 ± 0.00	00 ± 0.00	00 ± 0.00	00 ± 0.00	00 ± 0.00
Control treatment with Deltamethrin	100 + 0.00	00 ± 0.00	00 ± 0.00	00 ± 0.00	00 ± 0.00
Negative Control group with no treatment	00 ± 0.00	00 ± 0.00	00 ± 0.00	00 ± 0.00	00 ± 0.00

M.M. % ± S.E = mean mortality ± standard deviation. No mortalities in corresponding groups during the same exposure periods.

## Data Availability

The data presented in this study are available on request from the corresponding authors.
